# Decreased Cortisol and Pain in Breast Cancer: Biofield Therapy Potential

**DOI:** 10.1155/2015/870640

**Published:** 2015-06-11

**Authors:** Alice Running

**Affiliations:** College of Nursing No. 211, Montana State University, Bozeman, MT 59717-3560, USA

## Abstract

Breast cancer is one of the leading causes of cancer death among women of all races. Pain is a common symptom associated with cancer; 75–90% of cancer patients experience pain during their illness and up to 50% of that pain is undertreated. Unrelieved pain leads to increased levels of the stress hormone cortisol. The purpose of this study was to examine the impact of bioenergy on fecal cortisol levels for mice injected with murine mammary carcinoma 4T1 in two separate pilot studies. Using a multiple experimental group design, six to eight week old female BALB/c mice were injected with tumor and randomly assigned, in groups of 10, to daily treatment, every other day treatment, and no treatment groups. Five days after tumor cell injection, bioenergy interventions were begun for a period of ten consecutive days. Fecal samples were collected for each study and ELISA analysis was conducted at the end of both studies. For both studies, cortisol levels were decreased in the every other day treatment groups but remained high in the no treatment groups. Future studies utilizing bioenergy therapies on cortisol levels in a murine breast cancer model can begin to describe pain outcomes and therapeutic dose.

## 1. Introduction

Breast cancer is the most common cancer diagnosis for women in the United States and is one of the leading causes of cancer death among women of all races. According to the Center for Disease Control and Prevention, 211,831 women in the United States were diagnosed with breast cancer in 2009 and 40,676 women in the United States died from that diagnosis [1]. Pain is a common symptom associated with cancer; 75–90% of cancer patients experience pain during their illness and up to 50% of that pain is undertreated [2]. Pain that continues or is unrelieved (up to 50%) significantly impacts the patient and his/her family, making the diagnosis of cancer and progression of the disease even more difficult [2]. Unrelieved pain has been linked to increased levels of stress, as measured by the stress hormone cortisol [3], and increased levels of anxiety. Opioids are recognized by the WHO as the first line of treatment for cancer pain. Opioid therapies are effective and are relied upon heavily for management of cancer pain, but these therapies are not without side effects such as constipation, urinary retention, nausea, sedation, respiratory depression, myoclonus, delirium, sexual dysfunction, and hyperalgesia [3].

Complementary and Alternative Medicine (CAM) therapies are increasingly used by practitioners and patients alike to manage pain and are without the side effects known to be associated with opioids. In 1998, surveys on the use of CAM therapies by cancer patients were reported as high as 64% and as low as 7% [4]. According to the 2014 report by the National Cancer Institute at the National Institutes of Health, more than 50% of cancer patients use some form of CAM therapy [5]. CAM therapies that are found to be effective for pain related symptoms without the opioid side effects include acupuncture, biofield (Reiki, healing touch), massage, cranial stimulation, music therapy, and foot baths [6]. Acupuncture, biofield therapies, massage, and cranial stimulation remove or lessen blockages in pathways or channels that can lead to disruptions or disturbances in the flow of energy throughout the body. Once those blockages are removed, balance is restored to enable the body's innate tendency for healing to occur. More recently, psychoeducational interventions, Chinese herbal medicine, compound kushen injection, reflexology, lycopene, TENS, qigong, cupping, cannabis, homeopathy (Traumeel), and creative arts have also been found to have some positive impact on cancer pain [7–11]. These therapies impact the flow of energy as well and promote homeostasis, balance, and relaxation which is believed to impact pain and stress in a significant way. An area of special interest to the National Center for Complementary and Integrative Medicine (NCCIM) is alleviating pain and inflammation processes which makes continued research in this area of particular importance [7].

Women fear breast cancer more than any other disease and their levels of breast cancer-specific intrusions are related to their increased stress and perceived risk of breast cancer [12]. Cortisol is viewed as a physiological marker of stress [12, 13]. Chronic stress and cortisol deregulation can influence inflammation and immune function in ways that promote fatigue, depression, and risk of cancer recurrence [14, 15]. The stress of advancing cancer and management of it is associated with endocrine and immune dysfunction that has significant, negative consequences for host resistance to cancer progression [16].

At least a third of all patients who undergo treatment for cancer develop psychological morbidity, persisting throughout the disease continuum from suspicion to diagnosis, treatment, and beyond [17]. Others would say that psychological symptoms of distress are reported in as many as 41% of patients with a new diagnosis of breast cancer [18]. In an earlier study, 49.6% of women with early breast cancer were clinically anxious and 37.2% were clinically depressed in the first three months following surgery [19]. Later studies would show that 48% of women diagnosed with early breast cancer were clinically anxious and/or depressed in the first year [19].

The impact of anxiety for breast cancer patients has the potential to impact treatment response, decision making, and overall quality of life [17, 20, 21]. High levels of stress reactivity have recently been related to poor compliance with medical care and low quality of life scores for breast cancer survivors [22].

Stress-induced immunosuppression associated with diagnosis and treatment of breast cancer is well established [23]. High levels of stress in cancer patients have also been shown to negatively impact the immune system leading to elevations in proinflammatory cytokines and stress hormones. Studies have also demonstrated a relationship between stress and decreased immune measurements, even describing a relationship between the types of stress (acute versus chronic) involved and decreased function of immune systems [23–31]. Complementary alternative modalities, in particular bioenergy therapies, are showing promise in their abilities to mediate the impact of these dysfunctions.

Biofield modalities are putative in nature, meaning they are yet to be measured using Western empirical measures. Practices based on putative energy fields (also called biofields) generally reflect the concept that human beings are infused with subtle forms of energy. Practitioners are thought to modulate human biofields by identifying and then removing energy movement blockages. Some would argue that because this energy has never been demonstrated beyond a reasonable doubt, it lacks biological plausibility [32]. However patients continue to seek out these modalities and more sophisticated research methods are leading to a body of evidence that would support continued inquiry into the practice and results of bioenergy.

As reviewed above, stress and increased cortisol can negatively impact pain, immune function, and influence the formation and growth of cancers. Because stress hormones (like cortisol) are known to enhance tumor growth, angiogenesis, and invasion as well as impair cellular immune responses, a variety of cancer growth processes may be blunted and immunity supported by biofield therapies [17, 28, 32–37].

Published reviews regarding the utility of CAM therapies, to include bioenergy studies, have concluded that more research is needed to demonstrate, more specifically, the efficacy, meaning, and underlying mechanisms influenced by energy therapies and invited a more expansive view of what constitutes evidence. These reviews have recommended continued examination of specific biomarkers associated with stress and relaxation response systems to assist in determining the impact of biofield therapies on physiology and concluded that because of small sample size, high heterogeneity across studies, and high risk of bias for primary studies they could not recommend any CAM interventions for adult cancer pain. Murine models may be one mechanism that can begin to address some of these issues.

The current study explores the effects of biofield therapies on cortisol levels in mice injected with breast cancer cells. Aims of the study are as follows.

1. To determine whether mice injected with breast cancer cells and then treated with bioenergy demonstrate decreased levels of cortisol.
2. To determine if the results of the first study could be replicated with a different bioenergy practitioner.

## 2. Materials and Methods

### 2.1. Design

**Figure 1:**
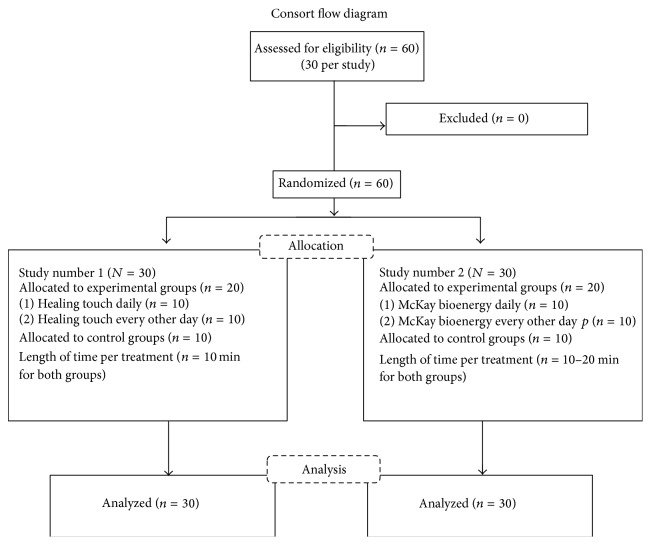
Consort Flow Diagram.

This study used a randomized, two-group repeated measures design. In two separate studies, two groups of interventional mice (daily healing touch and every other day healing touch) were compared to each other and to a group of untreated mice. Data were collected at two points: day 3 (before treatment) and day 12 (after treatment) (Figure 1).

### 2.2. Samples

For each study, thirty-six- to eight-week-old BALB/c mice (15–25 g) were obtained from Charles River Laboratories. The mice were housed in a ventilated barrier rack in a temperature controlled facility on a 12 h photoperiod. The mice were given food and water ad libitum. This research was conducted under a protocol approved by the Montana State University, Bozeman Institutional Animal Care and Use Committee (IACUC).

The 6-thioguanine-resistant 4T1 mouse mammary carcinoma cell line was obtained from the American Type Culture Collection (Rockville, MD) and grown in RPMI 1640 medium supplemented with 10% fetal bovine serum and 1% each of essential amino acids, L-glutamine, penicillin/streptomycin, and 10 mM HEPES. Cells were harvested for injection with 1X Trypsin/EDTA (Corning) to detach cells from the flask. Cells were then washed three times with Dulbecco's PBS and resuspended in DPBS for injection.

### 2.3. Procedure

Mice were randomly assigned to cages (five mice per cage, two cages per group, as per power analysis and literature review) when they arrived to the Animal Resource Center (ARC) and remained in those groups for the duration of the study. Five days after arrival, allowing time for acclamation to the ARC and their group, each mouse was injected with 100,000 cells of 4T1 murine mammary breast cancer tumor subcutaneously in 0.1 mL in the lower right mammary gland (day 1). Five days after injection (day 5), allowing time for tumor establishment, the intervention for each group began and continued for ten consecutive days.

Study one employed a certified healing touch practitioner. Group 1 received bioenergy treatment daily, group 2 received bioenergy treatment every other day, while group 3 received no treatment. The literature is not clear on duration of therapeutic treatment time. After consulting with an animal healing touch practitioner and reviewing the literature, it was decided to set a treatment time of 10 minutes per group.

Study two employed a bioenergy practitioner trained in The McKay method. Following the same procedure, group 1 received bioenergy treatment daily and group 2 received bioenergy treatment every other day, while group 3 received no treatment. For this study, treatment time lasted 10–20 minutes depending on the practitioner's daily assessment.

**Figure 2:**
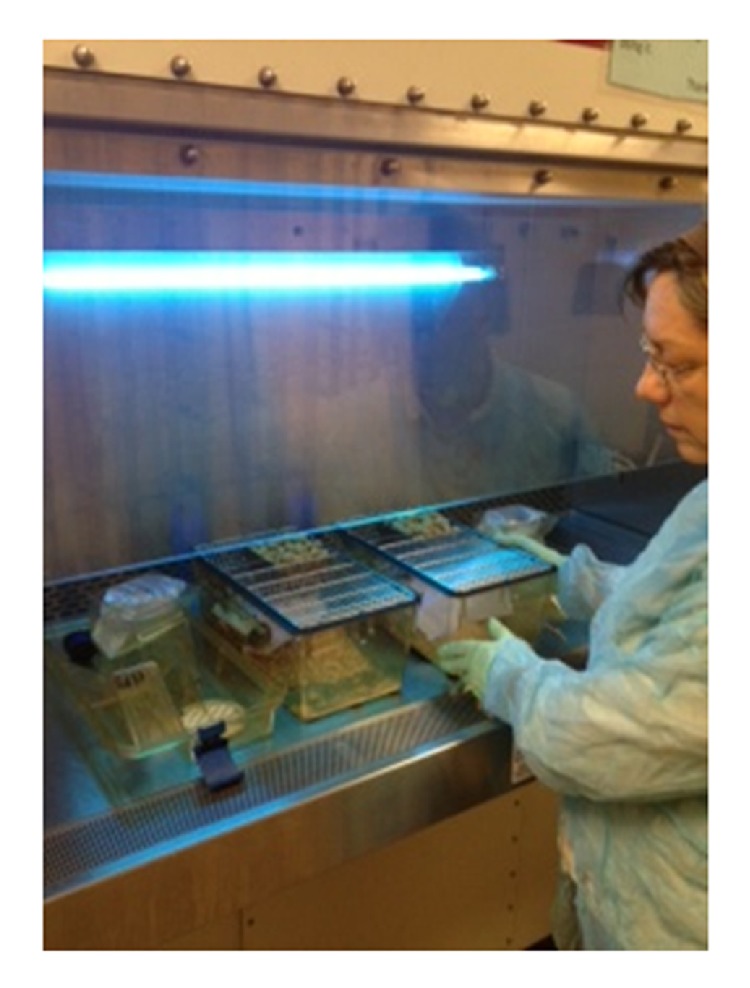
Practitioner setup for bioenergy intervention.

For each study, at the same time every day, the researcher would gown, enter the room where the mice were kept, glove, turn on the air exchange fan in the biosafety hood, move the appropriate cages from the rack, and place them under the hood. For each intervention group, the two cages (five mice in each cage) were placed side by side under the air exchange hood. Once the cages were placed under the hood, the plastic cover of the cage, along with the water which is kept inside the cage, was moved to the side of the hood leaving the metal slotted cover over each cage intact providing access to the mice for the practitioner. The researcher would then leave the room and the bioenergy practitioner would gown, enter the room, and prepare for the session (Figure 2).

Each healer prepared for the session by centering and aligning themselves, attuning to the mice and assessing their energy fields. The bioenergy practitioners then used a “hand scan” over the cages to determine levels of energy or auric field for each group. The practitioner would then “hold the field” to intensify energy to the mice and use “pain drain” to drain away irregularity from a specific or general area of the mouse bodies. “Hands in motion” would then be used to soothe and calm the field, and “hands still” was used to energize the spleen and adrenals. At no time did the practitioner come in physical contact with any of the experimental mice.

At the end of each treatment the practitioner would acknowledge the contribution of the mice, remove themselves from the mouse energy field, and leave the room. The researcher would return to the room, replace the water and plastic cover over each of the mice cages, and place the mice back in the same spot on the rack.

On day 3 and day 12 (three hours after receiving the bioenergy treatment) fecal samples were collected from each of the intervention and control mice. Stool samples were stored at −80 until analyzed. On day 15 of each study all of the mice were euthanized following IACUC protocol.

### 2.4. Extraction and Analysis of Fecal Cortisol

Fecal samples were weighed and then homogenized in 1 mL of 80% methanol. Samples were then shaken for 30 minutes on a multivortex. After shaking, samples were centrifuged for 10 min at 2500 g. An aliquot of each supernatant was then diluted (1 : 5) in assay buffer and analyzed by ELISA (Cortisol EIA kit, Enzo Life Sciences, Farmingdale, NY) as recommended by manufacturer.

### 2.5. Statistical Analyses

Statistical analyses were performed using Prism 4 (GraphPad Software, San Diego, CA). Fecal cortisol levels were compared by two-way ANOVA.

## 3. Results and Discussion

**Figure 3:**
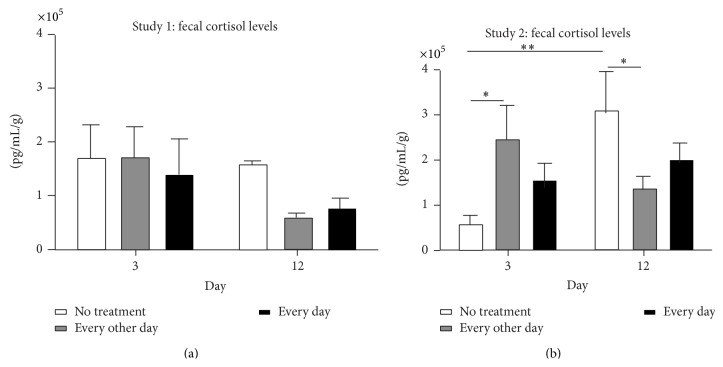
Fecal cortisol levels measured by ELISA. (a) Study 1 levels for untreated group (*n* = 2), every other day treatment group (*n* = 2), and daily treatment group (*n* = 2). (b) Study 2 levels for untreated group (*n* = 9), every other day treatment group (*n* = 10), and daily treatment group (*n* = 10). The data are expressed as mean +/− SEM. Statistical significance was measured by two-way ANOVA with Bonferroni posttest. ^∗^ *P* < 0.05, ^∗∗^ *P* < 0.01.

The goal of the first study was to determine if cortisol levels in mice with cancer could be influenced by healing touch. Fecal cortisol levels of pooled fecal matter from each cage of mice (2 cages/group) were examined at two time points. The first was two days after tumor injection and two days before the first treatment (day 3) and the second was near the end of the experiment, seven days after treatment began (day 12). At day 3, the fecal cortisol levels (measured as pg/mL/g) for the untreated ($\overset{-}{x}$ = 167958.2 and SD = 87684.2), every other day treatment ($\overset{-}{x}$ = 169258.9 and SD = 80096.4), and daily treatment ($\overset{-}{x}$ = 134221.5 and SD = 96297.9) groups were similar (Figure 3(a)). However, at day 12, fecal cortisol levels appeared to be lower in the every other day ($\overset{-}{x}$ = 55784.7 and SD = 13396.8) and daily ($\overset{-}{x}$ = 70997.9 and SD = 31523.8) treatment groups compared to the untreated group ($\overset{-}{x}$ = 154480.9 and SD = 9562.4). In addition, fecal cortisol levels appeared to be reduced between day 3 and day 12 in the every other day and daily treatment groups, but not the untreated group. The data was not statistically significant, likely due to the small number of samples per group, but these data provided preliminary evidence that cortisol levels in mice with cancer could be influenced by healing touch.

In study 2, we analyzed the cortisol levels of each mouse and enhanced our sample numbers (9 or 10 mice/group). We also wanted to determine if we could get similar results to the first study with a different practitioner. Before treatment, at day 3, fecal cortisol levels were higher in the every other day ($\overset{-}{x}$ = 246525.3 and SD = 229323.6) and daily treatment ($\overset{-}{x}$ = 151709.3 and SD = 118512.0) groups than the untreated group ($\overset{-}{x}$ = 55104.3 and SD = 61825.2) (Figure 3(b)). This difference was statistically significant between the untreated and every other day treatment group. However, after treatment, on day 12, fecal cortisol levels were actually lower in the every other day ($\overset{-}{x}$ = 134028.0 and SD = 86815.5) and daily treatment ($\overset{-}{x}$ = 196557.1 and SD = 122664.4) groups compared to the untreated group ($\overset{-}{x}$ = 307396.8 and SD = 255547.9). Again, this difference was statistically significant between the untreated and every other day treatment groups. Furthermore, fecal cortisol levels significantly increased in the untreated group between days 3 and 12, but not in the treated groups.

Bioenergy interventions were successful, in two separate pilot studies with two different practitioners, at reducing the levels of cortisol in female mice injected with mammary carcinoma 4T1 compared to untreated mice. These data are in agreement with what has been observed in clinical studies [11, 13–15, 20, 25–30]. While the first study results were not statistically significant, the direction of the relationship was compelling and the results from the second study further supported these preliminary findings.

In both studies, the mice receiving bioenergy treatments every other day had the lowest cortisol levels towards the end of the experiment. This suggests that there may be a dose effect to bioenergy interventions, but further studies would be needed to confirm this. Altogether, these data provide preliminary evidence that murine models may be a useful tool for exploration into the efficacy and mechanisms of action of bioenergy interventions on stress and possibly other physiological responses. Future studies that build on these findings are in line with NCCAMs call for the employment of animal models and methodology in basic and translational research to study the biological effects and mechanisms of action underlying CAM approaches [6].

Sample sizes for the two studies, though small, were typical for murine research and pilot studies. Cortisol levels are known to be variable, and in murine models there are no indicators available for normal cortisol levels or ranges, so normal values are not available for comparison.

## 4. Conclusions

Both of the aims developed for this study were accomplished. Through this research we were able to prove that mice injected with breast cancer cells and then treated with bioenergy demonstrated decreased levels of cortisol. Though the statistical significance for the first study was not as strong as the second, in both studies the cortisol levels decreased. These results would support earlier studies reporting the effectiveness of bioenergy on cortisol levels, stress, and immune function.

In regard to the second aim, a second bioenergy practitioner was able to achieve more significant results. As described in the methodology section, each practitioner employed the same techniques, but in the second study the practitioner was able to extend the time of the intervention. Because of the need for information on therapeutic dose and outcomes, the results of this study can be used in future protocol development.

The provision of complementary alternative modalities for women with breast cancer must include evidence either supporting or negating its impact. Incorporating a CAM modality such as bioenergy, with evidence from rigorous controlled studies, could have far reaching practice implications. Continued research into dose, length of treatment, and other variables which could impact efficacy as well as mechanisms of action is needed to optimize its use in the relief of pain and the provision of care for cancer patient. These studies can be difficult to perform in clinical settings. The results presented here, while limited in scope, provide evidence that larger scale studies in murine models could be used to address some of these questions and further examine the impact of bioenergy interventions in disease models. This knowledge could then potentially be translated into clinical studies to provide further evidence for practice. Because cancer pain will be experienced by as many as 90% of patients and because there are no known parameters for therapeutic dose for many CAM therapies, scientists and practitioners must work together developing and testing safe interventions to address this statistic. These pilot studies are a first step in that process.
